# Comparison of Two Species of *Notopterygium* by GC-MS and HPLC

**DOI:** 10.3390/molecules20035062

**Published:** 2015-03-19

**Authors:** Yaping Wang, Linfang Huang

**Affiliations:** Institute of Medicinal Plant Development, Chinese Academy of Medical Sciences & Peking Union Medical College, Beijing 100193, China; E-Mail: ypwang90@126.com

**Keywords:** *Notopterygium incisum*, *Notopterygium franchetii*, HPLC, GC-MS, chemometrics

## Abstract

Notopterygii Rhizoma et Radix (Qianghuo), including *Notopterygium incisum* Ting ex H. T. Chang (NI) and *Notopterygium franchetii* H. de Boiss (NF), is an important traditional Chinese medicine. Of these two plants, NI, is more commonly used and has a much higher price in the marketplace. To compare these two plants, a combination of gas chromatography-mass spectrometry (GC-MS) and high performance liquid chromatography (HPLC) was carried out, thus obtaining an overall characterization for both volatile and none-volatile compounds. Combined with hierarchical cluster analysis (HCA) and principal component analysis, GC-MS was successfully applied to distinguish NF and NI. The chemical constitutes of volatile oil in NI and NF were firstly compared in detail, and 1*R*-alpha-pinene, beta-pinene and 4-isopropyl-1-methyl-1,4-cyclohexadiene had great contribution to the discrimination. Fingerprints of 14 batches of Qinghuo samples were also established based on HPLC, and an obvious difference was found between the two species. The chromatographic fingerprints were further analyzed by similarity analysis and HCA. The present study is the first reported evaluation of two origins of Notopterygii Rhizoma et Radix by GC-MS and HPLC, which will facilitate quality control and its clinical application.

## 1. Introduction

Notopterygii Rhizoma et Radix (Qianghuo), a well-known traditional Chinese medicine, originates from the dried rhizome and root of *Notopterygium incisum* Ting ex H. T. Chang (NI) and *Notopterygium franchetii* H. de Boiss. (NF) according to the Chinese Pharmacopoeia (2010 edition) [1] and is famous for its diaphoretic, antifebrile, antirheumatic and anodyne characteristics in the treatment of rheumatism, headaches and colds [2,3]. Qianghuo, endemic to high altitude regions, is mainly distributed in Tibet, Sichuan, Qinghai and Gansu of China. Its main chemical constituents are coumarins, phenoloids and essential oils [4,5,6,7,8]. Pharmacological studies have indicated that coumarins, such as notopterol, bergapten and isoimperatorin, possess anti-inflammatory, analgesic, anti-cancer and anti-coagulant activities [9,10,11], and the volatile oils possess antifebrile, anodyne and anti-inflammatory activities [12]. However, the two origins of Qianghuo differ in terms of the cultivation altitude: NI usually grows at altitudes above of 3700 m, while NF grows at about 1600 m [13]. Due to overexploitation and habitat degeneration in recent years, Qianghuo is becoming endangered. In order to protect wildlife resources and to solve the market demand, quality control of Qianghuo is needed. Previous qualitative and quantitative analysis studies of Qinghuo have mainly focused on thin layer chromatography (TLC) [14], gas chromatography-mass spectrometry (GC-MS) [15], high performance liquid chromatography (HPLC) [16,17] and HPLC coupled with mass spectrometry (HPLC-MS) [18]. However, few studies have done a simultaneous analysis of the volatile and non-volatile components between NF and NI as a comparative study.

In order to qualitatively and quantitatively distinguish NF and NI, the combination of GC-MS and HPLC fingerprinting of multiple components of the two plants was conducted. The dataset obtained from GC-MS and HPLC was processed by principal component analysis (PCA) and hierarchical cluster analysis (HCA) to compare the difference of the two species.

## 2. Results and Discussion

### 2.1. Analysis of Volatile Compounds by GC-MS

**Figure 1:**
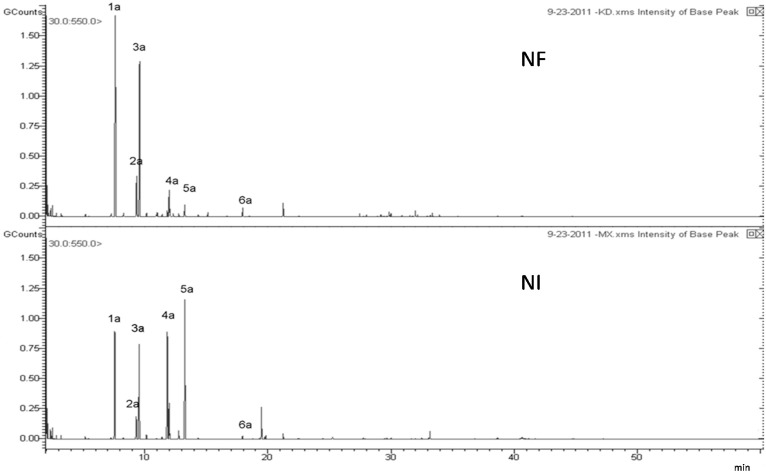
GC-MS chromatography profiling of *Notopterygium franchetii* (NF) and *Notopterygium incisum* (NI).

**Figure 2:**
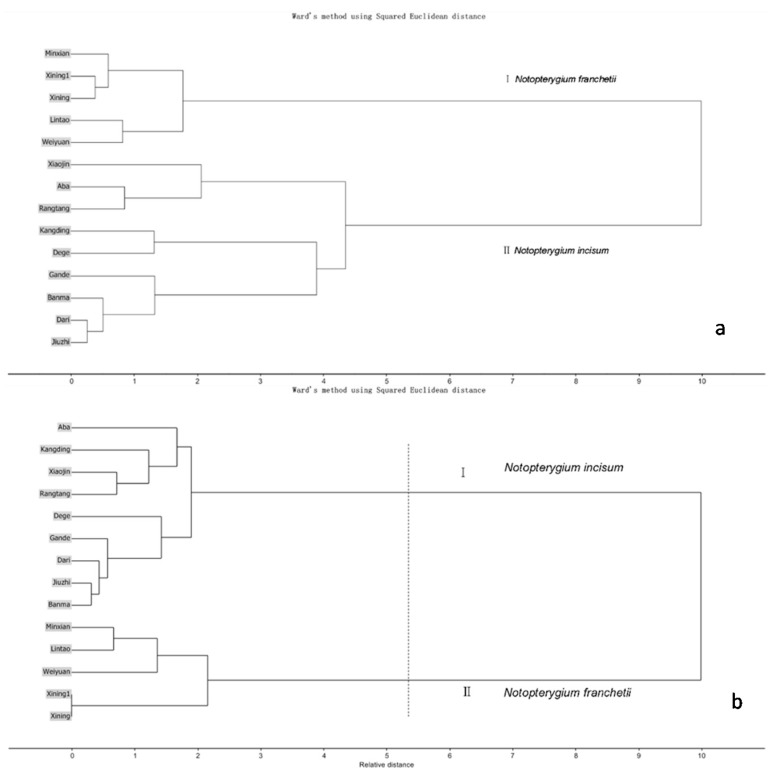
Hierarchical cluster analysis (HCA) results of 14 batches of Qianghuo samples based on GC-MS chromatograms (**a**) and HPLC fingerprints (**b**), respectively.

**Figure 3:**
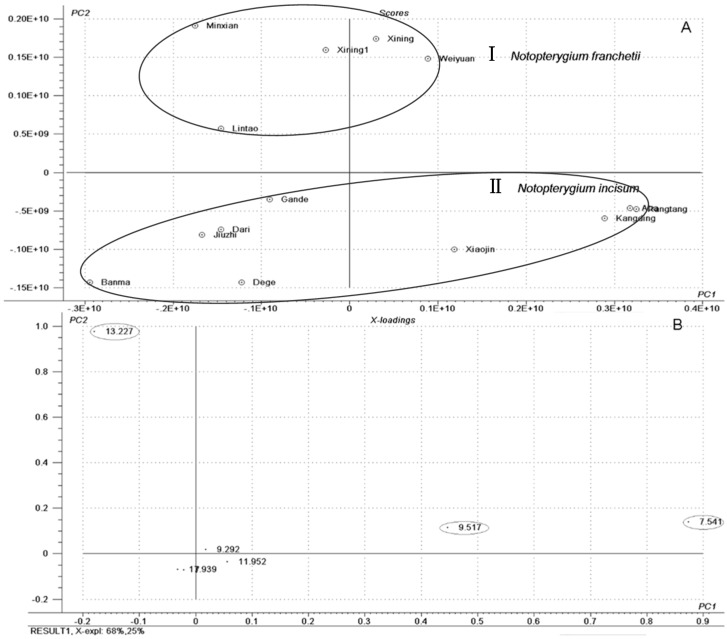
PCA scores plot (**A**) and loadings plot (**B**) for volatile oils in Qianghuo by GC-MS.

Volatile compounds comprise an important part of Qianghuo. For example, d-limonene shows multiple pharmacological effects, including antitussive, antibacterial and the inhibition of tumor [19,20]. The volatile oil compositions of Qianghuo were different [21]. In Figure 1 and Table 1, 39 compounds in NI and NF show differences in chromatography profiling and relative contents. The contents of 1*R*-alpha-pinene, beta-pinene, d-limonene and l-terpinen-4-ol were relatively higher in NI than in NF samples; whereas another two main components (4-isopropyl-1-methyl-1,4-cyclohexadiene and b-thujene) have a higher content in NF. Nine compounds were only detected in NI: bulnesol, 1-methyl-4-(1- methylethylidene)-cyclohexene, shyobunone, epicedrol, (6*R*)-1,1,5,9-tetramethylspiro [5.5]undeca-1,8-diene, guaiol, dehydroxy-isocalamendiol, r-eudesmol and 1-methyl-4-(1-methylethyl)-cyclohexene. Twelve compounds were only detected in NF: 3,7-dimethyl-1,3,6-octatriene, 2,6-dimethyl-2,4,6-octatriene, pentanoic acid, 2-methylbutyl ester, 2-butenoic acid, 2-methyl-, 3-methylbutyl ester, 1-isopropyl-2-methoxy-4-methylbenzene, 3-methyl-2-butenoic acid, alpha-bisabolol, octahydro-4,4,8,8-tetramethyl-4a,7-methano-4aH-naphth[1,8a-b]oxirene, apiol, 9-aristolene, agarospirol and *Z*-3-decen-1-yl acetate. In HCA and PCA, Cluster I was the NF samples, and Cluster II was the NI samples (Figure 2 and Figure 3). This results present that NI and NF could be separated based on volatile oils by GC/MS. The loading plot of PCA (Figure 3) indicated that 1*R*-alpha-pinene, beta-pinene and 4-isopropyl-1-methyl-1,4-cyclohexadiene had a great contribution toward the discrimination of NI and NF. 

**Table 1:** Compounds identified by GC-MS.

Peak No.	t_R_/min	Name		Formula	CAS	Area/% in NI	Area/% in NF
1a	7.612	1*R*-alpha-pinene		C_10_H_16_	7785-70-8	34.61	8.86
2a	9.331	b-thujene		C_10_H_16_	28634-89-1	3.91	4.49
3a	9.576	beta-pinene		C_10_H_16_	18172-67-3	25.79	19.89
4a	11.98	d-limonene		C_10_H_16_	5989-27-5	9.59	4.28
5a	13.243	4-isopropyl-1-methyl-1,4-cyclohexadiene		C_10_H_16_	99-85-4	2.18	21.23
6a	17.949	l-terpinen-4-ol		C_10_H_18_O	20126-76-5	1.63	1.02
7	5.187	ethylbenzene		C_8_H_10_	100-41-4	0.15	0.19
8	7.237	3-thujene		C_10_H_16_	2867-5-2	0.49	0.22
9	8.283	camphene		C_10_H_16_	79-92-5	0.61	0.4
10	10.146	beta-myrcene		C_10_H_16_	123-35-3	0.4	0.54
11	10.933	alpha-phellandrene		C_10_H_16_	99-83-2	25.79	0.9
12	11.034	3-carene		C_10_H_16_	13466-78-9	10.56	0.48
13	11.409	2-carene		C_10_H_16_	554-61-0	0.62	0.48
14	11.828	1-methyl-2-(methylethyl)-benzene		C_10_H_14_	527-84-4	0.92	11.38
15	12.041	4-methylene-1-(1-methylethyl)-bicyclo[3.1.0]hexane		C_10_H_16_	3387-41-5	0.18	1.11
16	12.766	3,7-dimethyl-1,3,6-octatriene		C_10_H_16_	3338-55-4	0	4.52
17	14.181	1-methyl-4-(1-methylethyl)-cyclohexene		C_20_H_36_	34363-01-4	0.1	0
18	14.325	2,6-dimethyl-2,4,6-octatriene		C_10_H_16_	673-84-7	0	0.38
19	14.333	1-methyl-4-(1- methylethylidene)-cyclohexene		C_10_H_16_	586-62-9	0.56	0
20	15.155	pentanoic acid, 2-methylbutyl ester		C_10_H_20_O_2_	55590-83-5	0	0.25
21	18.295	2-butenoic acid, 2-methyl-, 3-methylbutyl ester		C_10_H_18_O_2_	66917-62-2	0	1.21
22	19.478	1-isopropyl-2-methoxy-4-methylbenzene		C_11_H_16_O	1076-56-8	0	2.01
23	19.796	3-methyl-2-butenoic acid		C_11_H_16_O_2_	-	0	0.93
24	21.241	(1*S*,2*R*,4*S*)-bicyclo[2.2.1]heptan-2-ol,1,7,7-trimethyl-, 2-acetate		C_12_H_20_O_2_	5655-61-8	1.86	1.21
25	25.281	*Z*-3-decen-1-yl acetate		C_12_H_22_O_2_	81634-99-3	0	2.78
26	29.173	shyobunone		C_15_H_24_O	-	0.34	0
27	29.845	epicedrol		C_15_H_26_O	19903-73-2	1.07	0
28	29.981	d-cadinene		C_15_H_24_	483-76-1	1.13	1.46
29	30.214	octahydro-4,4,8,8-tetramethyl-4a,7-methano-4aH-naphth[1,8a-b]oxirene		C_15_H_24_O	67999-56-8	0	0.56
30	30.249	(6*R*)-1,1,5,9-tetramethylspiro [5.5]undeca-1,8-diene	C_15_H_24_		19912-83-5	0.77	0
31	31.974	guaiol	C_15_H_26_O		489-86-1	0.64	0
32	32.162	dehydroxy-isocalamendiol	C_15_H_24_O		-	0.4	0
33	32.208	r-eudesmol	C_15_H_26_O		473-16-5	0.87	0
34	32.514	apiol	C_12_H_14_O_4_		523-80-8	0	0.75
35	32.717	1*R*,4*S*,7*S*,11*R*-2,2,4,8-tetramethyltricyclo [5.3.1.0(4,11)]undec-8-ene	C_15_H_24_		-	0.15	0.65
36	33.026	9-aristolene	C_15_H_24_		6831-16-9	0	0.68
37	33.163	agarospirol	C_15_H_26_O		1460-73-7	0	4.75
38	33.36	bulnesol	C_15_H_26_O		22451-73-6	0.29	0
39	33.741	alpha-bisabolol	C_15_H_26_O		515-69-5	0	1.94

Notes: a The compound contents are high, both in NI and NF; CAS: Chemical Abstracts Service.

### 2.2. Analysis of Non-Volatile Compounds by HPLC

The simultaneous determination of the four marker compounds in Qianghuo, including ferulic acid, bergapten, notopterol and isoimperatorin, was analyzed. The results of these two species from different locations are shown in Table 2. Notopterol and isoimperatorin are the chemical indicators in the Pharmacopeia of China (2010 edition) [1] of Qianghuo. The content of notopterol was higher in the NI samples than in NF samples; while the content of isoimperatorin was higher in NF than in NI samples. However, the differences of content in notopterol and isoimperatorin were not obvious in the same species. Therefore, the contents of these two compounds can be applied to distinguish the two species of Qianghuo. Bergapten presents pharmacological effects, including photosensitization, anticancer and inactivation of infectious pathogens and leukocytes in platelets and plasma [22], as a medicinal compound. It could not be detected in NI, which is from Dari county, Qinghai province. Therefore, bergapten was inappropriate as a marker compound in evaluating quality. Finally, the contents of bergapten and ferulic acid have no significant differences in NI and NF.

**Table 2:** Sample list: contents of the 4 compounds in Qianghuo (14 batches) and fingerprint similarities.

No.	Samples	Location	Species	Content (mg/g)				Similarities
				(1)	(2)	(3)	(4)	
1	Xining *	Xining city, Qinghai province	NF	0.77	0.16	0.36	33.42	0.975
2	Xining *	Xining city, Qinghai province	NF	0.77	0.16	0.35	33.41	0.972
3	Weiyuan	Weiyuan county, Gansu province	NF	0.21	1.05	0.17	13.12	0.957
4	Minxian	Min county, Gansu province	NF	1.38	1.12	0.58	20.07	0.994
5	Lintao	Lintao county, Gansu province	NF	1.08	0.94	1.92	22.08	0.972
6	Rangtang	Rangtang county, Sichuan province	NI	1.23	0.18	12.83	5.36	0.955
7	Xiaojin	Xiaojin county, Sichuan province	NI	0.68	0.76	8.24	1.29	0.887
8	Aba	Aba county, Sichuan province	NI	1.13	0.01	7.89	1.89	0.819
9	Jiuzhi	Jiuzhi county , Qinghai province	NI	0.66	0.02	11.28	2.96	0.983
10	Gande	Gande county , Qinghai province	NI	0.61	0.11	9.37	3.94	0.977
11	Dari	Dari county , Qinghai province	NI	0.57	ND	8.14	3.03	0.988
12	Banma	Banma county , Qinghai province	NI	0.56	0.07	10.40	3.20	0.981
13	Kangding	Kangding county, Sichuan province	NI	1.97	0.28	22.79	4.72	0.970
14	Dege	Dege county, Sichuan province	NI	0.91	0.11	13.88	4.21	0.933

Notes: (1) ferulic acid; (2) bergapten; (3) notopterol; (4) isoimperatorin; ND: not detected. * S1: “tiaoqiang”; * S2: “datouqiang”, * S1 and * S2 are two different kinds of *Notopterygium franchetii*.

### 2.3. HPLC Fingerprint Analysis

**Figure 4:**
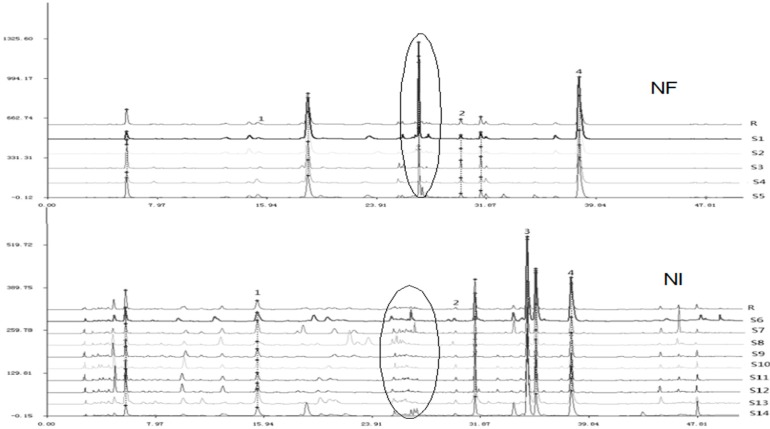
HPLC fingerprints of 14 batches of Qianghuo samples (R, reference chromatogram).

The chromatograms of the NF and NI samples (14 batches) are shown in Figure 4. Four main compounds were recognized by comparing the retention times and UV spectra with standards of ferulic acid, bergapten, notopterol and isoimperatorin. The profiles with reasonable heights and separation were assigned as “characteristic peaks” for the distinction of the two species. The correlation coefficient of similarity between each chromatographic profile of Qianghuo and the reference chromatogram, a representative standard fingerprint/chromatogram for a group of chromatograms, were calculated (Table 2), respectively. The correlation coefficients of the five batches of NF were higher than 0.95, and the correlation coefficients of the nine batches of NI were higher than 0.80. In Figure 4, the samples of NI and NF from different locations demonstrate certain differences. The differences among the chemical profiles of NI from different locations are bigger than of NF. This indicates that environmental factors may have more influence on the chemical composition of NI. These two species also show differences in the circled peak (t_R_ 26.77 min) and Peak 3 (t_R_ 35.25). Besides, the chromatograms of the NI and NF samples can be divided into two groups by HCA (Figure 2). The HPLC fingerprint result of Qianghuo is significant for identifying the two species, because of their similar appearance, and provides scientific information about the species.

## 3. Experimental Section

### 3.1. Plant Material

**Figure 5:**
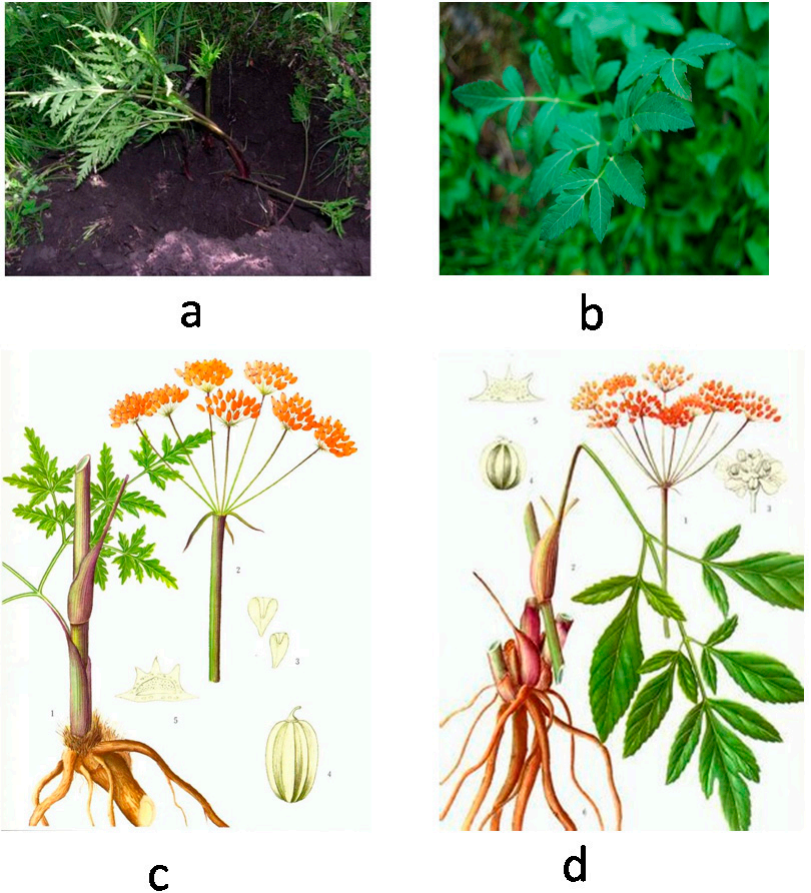
Pictures of NI (**a**,**c**) and NF (**b**,**d**).

In the present study, 14 batches of samples were collected from China in October, 2010 (Table 2). Among them, 5 batches were identified as NF, and 9 batches were identified as NI by Prof. Lin Yulin (Institute of Medicinal Plant Development, Chinese Academy of Medical Sciences, Beijing, China). The pictures of NI and NF are shown in Figure 5. Voucher specimens were deposited in the Herbarium of Institute of Medicinal Plant Development, Chinese Academy of Medical Sciences (Beijing, China).

### 3.2. Chemicals and Reagents

**Figure 6:**
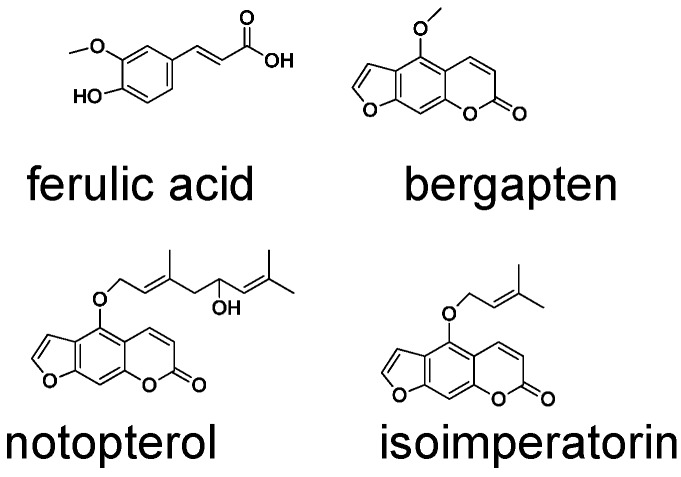
Chemical structures of the standard compounds.

Ferulic and bergapten were purchased from the National Institute for Control of Pharmaceuticals and Biological Products (Beijing, China), and notopterol and isoimperatorin were purchased Tongtian Biological Co., Ltd. (Shanghai, China). Their structures are shown in Figure 6. The purity of the compounds was higher than 98%, as determined by GC-MS or HPLC. Methanol (HPLC grade), acetic ether and acetonitrile were purchased from Fisher (Fisher Scientific, Fairlawn, NJ, USA); phosphoric acid (analytical grade) was purchased from Sigma (Sigma-Aldrich, St Louis, MO, USA). Deionized water was prepared by passing distilled water through a Milli-Q system (Millipore Corp., Bedford, MA, USA).

### 3.3. Apparatus and Chromatographic Conditions

GC-MS was performed with an Agilent 6890 gas chromatograph (Agilent Co., Palo Alto, CA, USA) coupled with a Varian 300 triple quadruple mass spectrometer (Varian Inc., Walnut Creek, CA, USA). The column was initiated at 50 °C at a rate of 2 °C/min to 70 °C, then to 118 °C at 5 °C/min. Later, the temperature was programmed from 118 °C to 128 °C at 2 °C/min and kept for 2 min; and finally, the temperature was increased to 240 °C at 8 °C/min [15]. The temperature of the split injector was 250 °C, and the split ratio was 15:1. High-purity helium (99.99%) was used as the carrier gas at a flow rate of 30 mL/min. The spectrometer was operated in the electron-impact mode, and the ionization and photomultiplier voltage energy were 70 eV and 1.25 kV, respectively. The ion source temperature was 230 °C. The scan rate was 3.8 scan/s, from 30 to 550 amu, with a solvent delay of 2 min.

HPLC analysis was performed on a Waters 1525 system, including a 717 automatic sampler, a column oven, an on-line degasser, a binary gradient pump, a 2487 ultraviolet detector and the Waters chromatography working station Breeze 2 (Waters, Milford, MA, USA). A reverse-phase XBridge C18 column (250 mm × 4.6 mm × 5 μm, Waters, Milford, MA, USA) was used for separating, and the column temperature was kept constant at 35 °C. The mobile phase consisted of (A) acetonitrile and (B) water with 0.01% phosphoric acid (H_3_PO_4_). The gradient program cwas as follows: 0–13 min (13%–17%, B), 13–20 min (17%, B), 20–22 min (17%–44%, B), 22–31 min (44%–57%, B), 31–38 min (57%, B), 38–45 min (57%–100%, B), 45–50 min (100%, B) [22,23]. The flow rate was 1 mL/min, and the injection volume was 10 µL. The detection wavelength was set at 310 nm.

### 3.4. Sample Preparation

For GC-MS analysis, the essential oils were obtained via water-steam hydrodistillation [24]. Ninety grams of the pulverized sample (through a 24-mesh sieve) were weighed accurately and then refluxed with water. The obtained volatile oil was dried using sodium sulfate anhydrous (Na_2_SO_4_) and kept at 4 °C, and the anhydrous volatile oils were diluted with acetic ether before injection. Then, 1 µL of the diluted volatile oil was injected into the GC-MS system for each analysis.

For HPLC analysis, 200 mg of the pulverized sample (through a 65-mesh sieve) were weighed accurately and macerated in 25 mL of methanol. The sample was then extracted for 30 min in an ultrasonic bath at 20 °C, and the loss of weight due to the evaporation of solvent was replenished with methanol. The supernatant was filtered through a 0.22-μm membrane. Then, 10 μL of the filtrate were injected into the HPLC system.

### 3.5. Data Processing and Multivariate Analysis

Comprehensive fingerprint profiles were performed by the “Similarity Evaluation System for Chromatographic Fingerprint of TCM” software (SESC-TCM, Version 2004 A, Chinese Pharmacopoeia Commission) [25,26]. The software can export the reference chromatogram based on correlation coefficients (R_1_); the R_1_ was calculated with median or average data and was expressed as follows [27]:

$$R_{1}=\frac{\sum_{i=1}^{n}(x_{i}-\overset{ˉ}{x})(y_{i}-\overset{ˉ}{y})}{\sqrt{\sum_{i=1}^{n}{\left(x_{i}-\overset{ˉ}{x}\right)}^{2}\sum_{i=1}^{n}{\left(y_{i}-\overset{ˉ}{y}\right)}^{2}}}\left(i=1,2,3,\ldots ,n\right)$$

The similarities of the samples are shown in Table 2. The HPLC fingerprint of samples were analyzed by HCA (Unscrambler^®^ X, 10.1 trial version, CAMO Software AS, Oslo, Norway) based on Ward’s method and the squared Euclidean distance. The identification of volatile oils in GC-MS was based on the NIST02 library, and relative height peaks of the identified compounds were analyzed by HCA (Table 1). The dataset of the volatile oil relative peak area was calculated via mean normalization.

## 4. Conclusions

In the present study, simple and accurate GC-MS and HPLC methods for the determination of 39 volatile and four non-volatile compounds were used to analyze NI and NF, two origins of Qianghuo. The GC-MS comparative analysis results of NI and NF showed that 1*R*-alpha-pinene, beta-pinene and 4-isopropyl-1-methyl-1,4-cyclohexadiene had a great contribution to the discrimination. Fourteen batches of Qianghuo from different locations were also assessed and distinguished by HPLC fingerprint analysis. With the combination of HPLC-HCA and GC/MS-HCA/PCA, the two species were found to differ significantly in both volatile and non-volatile compounds. Considering the differences in the chemical compositions, further investigation and comparison about the biological and pharmacological activities should be done to ensure the quality and efficacy of Qianghuo. Because NI grows at a higher altitude than NF, which means it is harder to cultivate, the price of NI is much higher than NF in the marketplace. Compared to the research conducted on NI, there exists little comparable research in the field of NI, and more work on NF is thus required in future studies.
